# Involvement of SET in the Wnt signaling pathway and the development of human colorectal cancer

**DOI:** 10.3892/ol.2014.1866

**Published:** 2014-02-11

**Authors:** LEI DONG, JIANJUN ZHU, XIAOXIA WEN, TINGTING JIANG, YAO CHEN

**Affiliations:** Department of Anatomy, Basic Medical and Forensic Medical Institute, Sichuan University, Chengdu, Sichuan 610041, P.R. China

**Keywords:** colorectal adenocarcinoma, Wnt signaling pathway, SET, RNAi

## Abstract

The SET oncoprotein is involved in cancer progression by modulating multiple cellular processes, including the inhibition of the tumor suppressor, protein phosphatase 2 (PP2A). Based upon these multiple activities, we hypothesized that targeted inhibition of SET is likely to have multiple discrete and measurable effects on cancer cells. In the present study, the mRNA expression levels of *SET*, *PP2A* and *β*-*catenin* were examined in 31 pairs of human colorectal adenocarcinoma tissues and corresponding adjacent normal colorectal tissues by quantitative real-time polymerase chain reaction (qPCR). A small interfering RNA targeting *SET* was transfected into the human colon carcinoma cell lines, LS174T and SW480. The mRNA levels of *SET, PP2A, β*-*catenin*, *c-Myc*, *E-cadherin* and *p53* were determined by qPCR analysis and the protein levels of SET, c-Myc, PP2A and β-catenin were examined by western blot analysis. mRNA expression levels of *SET* and *β-catenin* were found to be elevated in 22 (70.9%) samples, while PP2A expression levels were upregulated in eight (25.8%) samples. In addition, the knockdown of *SET* mRNA expression caused the upregulation of *PP2A* and *c-Myc* in the two cell lines, whereas *β-catenin, E-cadherin* and *p53* mRNA expression was downregulated. Consistent with these results, the protein expression of β-catenin and c-Myc was found to be downregulated, whereas PP2A was upregulated at the protein level. Based on these results, we proposed that SET is essential in the carcinogenesis of human colorectal adenocarcinoma. In addition, it is suggested that SET promotes carcinogenesis through regulation of the Wnt signaling pathway.

## Introduction

Colorectal adenocarcinoma is one of the most common malignancies and its prevalence has recently been increasing. With the second highest incidence among all types of cancer, it is also the second most common cause of cancer-related mortality worldwide (1,2). The carcinogenesis of colorectal adenocarcinoma is a multi-step process that involves a number of genomic alterations. Investigating the molecular mechanisms of this process may aid in cancer prevention, early diagnosis and the development of effective treatments.

Employing a cDNA subtractive library and cDNA microarray technology, our previous study identified 86 cDNA sequences differentially expressed between human colorectal adenocarcinoma and paratumor normal colorectal tissues (3). In particular, the present study focused on one differentially expressed sequence (GenBank accession number, ES274071), identified its full-length cDNA and determined it to be the *SET* gene. The *SET* gene is a largely nuclear protein, located on chromosome 9q34 centromeric to c-abl and Nup214, which is fused to Nup214 (also termed CAN), apparently as a result of translocation (4,5). It is predicted to encode a 27-amino acid protein with a molecular weight of 39 kDa. SET is overexpressed and has been found to play a role in acute myeloid leukemia oral carcinoma and ovarian cancer (6–9), but its involvement in the development of colorectal adenocarcinoma remains unknown. In order to determine whether SET is involved in the carcinogenesis of colorectal adenocarcinoma and the Wnt signaling pathway, the mRNA expression of *SET*, protein phosphatase 2 (*PP2A)* and β-*catenin* in human colorectal adenocarcinoma and paratumor normal tissues was determined by quantitative real-time polymerase chain reaction (qPCR) analysis. In addition, *SET* expression was knocked down by transient transfection of small interfering RNA (siRNA) targeting *SET,* and the intracellular changes were investigated at the mRNA and protein level.

## Materials and methods

### Tissue specimens

Colorectal adenocarcinoma and paratumor normal tissues were obtained from 31 patients at the West China Hospital of Sichuan University (Chengdu, China). The sample acquisition was approved by the Medical Research Ethics Committee of Sichuan University (Chengdu, China) and written informed consent was obtained from all patients. The absence of cancer cells in the collected normal tissues was confirmed through pathological examination, and these tissues were snap-frozen and stored in liquid nitrogen prior to subsequent analysis.

### Antibodies

The antibodies used were as follows: Anti-SET (Santa Cruz Biotechnology, Inc., Santa Cruz, CA, USA), -PP2A, -β-catenin, -c-Myc and -β-actin (Signalway Antibody Co., Ltd., College Park, MD, USA); and anti-rabbit or -mouse horseradish peroxidase-conjugated secondary antibodies (Santa Cruz Biotechnology, Inc.). Detection was performed using a Chemiluminescent Western detection kit (Thermo Fisher Scientific, Waltham, MA, USA).

### qPCR of tissue RNA extracts

Total RNA from tumor and paratumor normal tissues was isolated using the TRIzol RNA isolation reagent (Invitrogen Life Technologies, Carlsbad, CA, USA) according to the manufacturer’s instructions. cDNA was synthesized using the M-MuLV reverse transcriptase kit (Fermentas International Inc., Burlington, ON, Canada) and qPCR was performed using SYBR Premix Ex Taq (Takara Bio, Inc., Shiga, Japan). The amplification reaction was conducted according to the manufacturer’s instructions and associated international standards (10,11). qPCR primers used to amplify the *SET*, *PP2A* and *β*-*catenin* genes are shown in Table I.

### Cell culture

Human colon adenocarcinoma cell lines, LS174T and SW480 (American Type Culture Collection, Manassas, VA, USA), were cultured in DMEM, with 10% fetal bovine serum, penicillin (100 U/ml) and streptomycin (0.1 mg/ml) in a humidity controlled 37°C incubator with 5% CO_2_ atmosphere.

### Knockdown of SET mRNA expression and transient transfection

The knockdown of *SET* expression was conducted via the transfection of siRNA at a concentration of 100 nM. Cells were transiently transfected with a pool of six siRNA duplexes targeting *SET* (cat. no. 10621164446; Guangzhou RiboBio Co., Ltd., Guangzhou, China). LS174T and SW480 cells were seeded at a density of 2.3×10^6^ cells/well in 100-mm dishes. Transient transfection of siRNA targeting *SET* or control oligonucleotides into LS174T and SW480 cells was performed using Lipofectamine 2000 (Invitrogen Life Technologies).

### qPCR analysis of siRNA-transfected cells

The knockdown cells lines of *SET* mRNA were assessed by qPCR. According to the manufacturer’s instructions, total RNA from the tumor and matched normal tissues were isolated using the TRIzol RNA isolation reagent (Invitrogen Life Technologies). The total RNA was reverse-transcribed using the M-MuLV Reverse Transcriptase kit (Fermentas International Inc.) and qPCR was performed using SYBR Premix ExTaq (Takara). qPCR was performed on the the tumor and matched normal tissues of patients with colorectal cancer. The mean threshold cycle (Ct) and standard error were calculated from individual Ct values obtained from three replicates per specimen. The normalized mean Ct was calculated as ΔCt by subtracting the mean Ct of GAPDH from the target genes. ΔΔCt was calculated as the difference between the control ΔCt and the values obtained for each sample. The n-fold change in gene expression, relative to the untreated controls, was calculated using the 2^−ΔΔCt^ method (12). qPCR primers used for the amplification of these genes are shown in Table I.

### Western blot analysis

Protein extracts of the siRNA-transfected cells were acquired by lysing cells in ice-cold radioimmunoprecipitation assay buffer (Beyotime Institute of Biotechnology, Haimen, China) according to the manufacturer’s instructions. The total protein concentration was determined using an Enhanced BCA Protein assay kit (Beyotime Institute of Biotechnology). Equal protein samples were then denatured and separated by sodium dodecyl sulfate-polyacrylamide gel electrophoresis (Millipore, Billerica, MA, USA), transferred to nitrocellulose membranes (Millipore) and immunoblotted with anti-SET, -PP2A, -β-catenin, -c-Myc and -β-actin antibody.

### Statistical analysis

Data are presented as the means ± standard deviation of three or more replicate experiments. Statistical analysis was performed by Student’s t-test, Fisher’s exact probability test and analysis of variance (ANOVA) using SPSS 19.0 software (SPSS, Inc., Chicago, IL, USA). P<0.05 was considered to indicate a statistically significant difference.

## Results

### mRNA expression levels of SET, PP2A and β-catenin in human colorectal adenocarcinoma tissues

**Figure 1:**
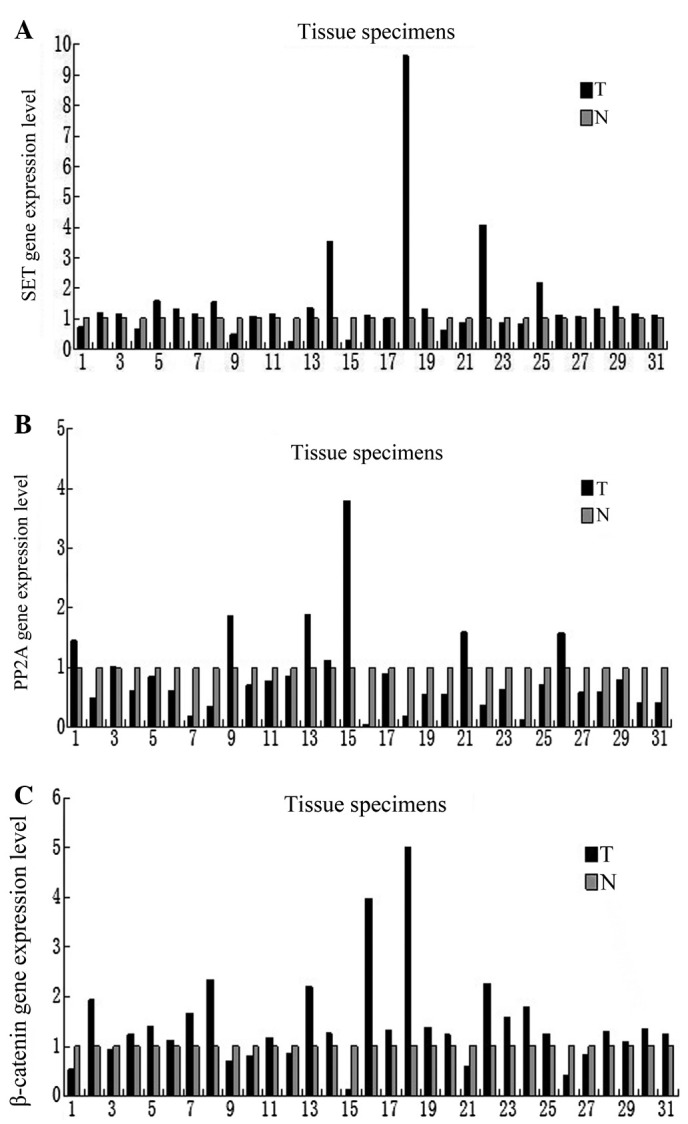
Quantitative real-time polymerase chain reaction analysis of (A) *SET* (B) *PP2A* and (C) *β-catenin* mRNA expression in human colorectal adenocarcinoma (T) and paratumor normal (N) samples. GAPDH was employed as a loading control. Numbers present individual patients. PPA2, protein phosphatase 2.

The expression of *SET*, *PP2A* and *β*-*catenin* at the mRNA level was tested in non-malignant and malignant tissues, and *SET* was found to be markedly elevated in 70.9% of the tumor samples (22 out of 31 samples; Fig. 1A). *PP2A* was upregulated in 25.8% of the tumor samples (eight out of 31; Fig. 1B), while *β-catenin* was upregulated in 70.9% of the tumor samples (22 out of 31; Fig. 1C) compared with the paratumor normal tissues. No significant correlation was found between the mRNA expression levels of *SET*, *PP2A* and *β*-*catenin* and gender, age, Dukes’ stage and differentiation degree by Fisher’s exact probabilities test (Table II).

### mRNA expression levels of SET, PP2A, β-catenin, p53, c-Myc and E-cadherin in LS174T and SW480 cell lines following SET knockdown

The mRNA expression levels of *SET* were analyzed in the LS174T *SET*-knockdown cell line and found to be significantly lower than those in the LS174T cell line. The reduction rate was 69.43%, which was statistically significant as shown by the ANOVA test (P=0.002). In addition, the *SET* expression levels in the SW480 *SET-*knockdown cell line were significantly lower than those in the SW480 cell line. The reduction rate was 75.68%, which was statistically significant as shown by the ANOVA test (P<0.001). Compared with the control cells, the mRNA expression levels of *p53, β-catenin* and *E-cadherin* were reduced in the LS174T and SW480 *SET*-knockdown cell lines. The percentage reduction in gene expression in the LS174T cells was 35.38, 62.89 and 26.29%, respectively, and all these values were found to be statistically significant by ANOVA when compared with the blank control group and the negative control groups (P=0.001, P=0.001 and P=0.048, respectively). The respective decrease rates in the SW480 cells were 23.16, 63.14 and 47.88%, and all these values were found to be statistically significant by ANOVA when compared with the blank control and the negative control groups (P=0.001, P<0.001 and P=0.001, respectively).

**Figure 2:**
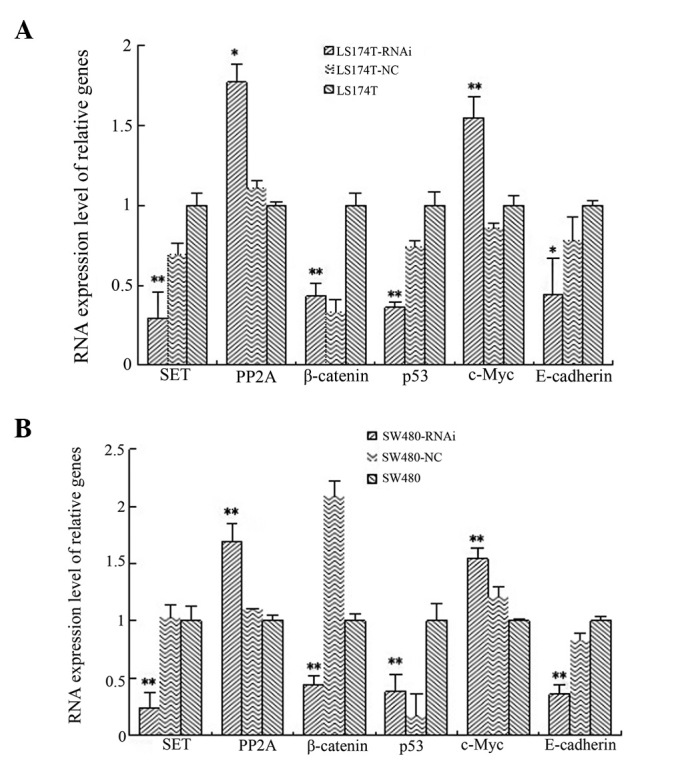
(A) qPCR analysis showing that *SET* knockdown in LS174T cells caused an increase in *PP2A* and *c-Myc* expression and reduced the expression levels of *SET*, *β*-*catenin*, *p53* and *E-cadherin.* (B) qPCR analysis demonstrating that *SET* knockdown in SW480 cells led to *PP2A* and *c-Myc* being upregulated while the relative mRNA expression levels of *SET*, *β-catenin, p53* and *E-cadherin* were reduced. GAPDH was used as a loading control. Statistical differences are ^*^P<0.05 and ^**^P<0.01 when compared with the blank control and negative control groups respectively, analysis of variance. Data are presented as the means ± standard deviation of triplicate cultures. qPCR, quantitative real-time polymerase chain reaction; PPA2, protein phosphatase 2.

The expression of *PP2A* and *c-Myc* were found to be elevated in the *SET-*knockdown cells. The increase rates in gene expression were 70.53 and 46.40%, respectively, in LS174T cells (P=0.015 and P=0.002, respectively), whereas the increase rates were 61.33 and 37.55%, respectively, in SW480 cells (P=0.001 and P<0.001, respectively) (Fig. 2).

### Knockdown of SET alters the protein expression of β-catenin, c-Myc, SET and PP2A in LS174T and SW480 cell lines

**Figure 3:**
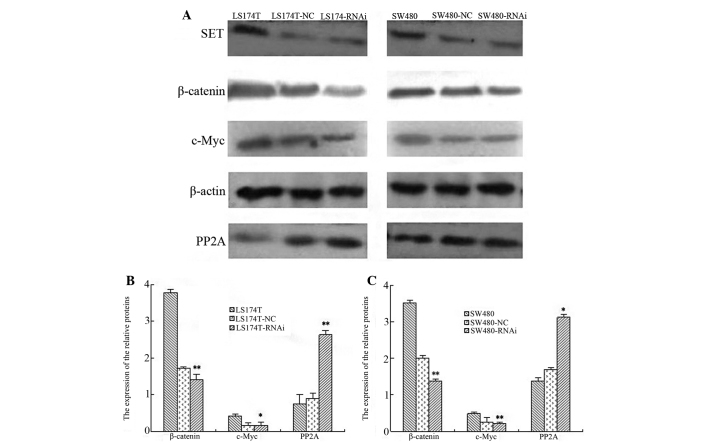
Downregulation of SET leads to changes of the indicated protein levels in colorectal adenocarcinoma cells using western blot analysis. (A) Protein expression levels of β-catenin, c-Myc and PP2A in the LS174T, LS174T^NC^, LS174T^RNAi^, SW480, SW480NC and SW480^RNAi^ cells. β-catenin and c-Myc were downregulated, whereas PP2A was upregulated in (B) LS174T^RNAi^ and (C) SW480^RNAi^ cells. β-actin was used as a loading control. ^*^P<0.05 and ^**^P<0.01 when compared with the blank control and negative control groups respectively. Data are presented as the means ± standard deviation of triplicate cultures. RNAi, RNA interference; PPA2, protein phosphatase 2.

The knockdown of *SET* led to the reduced protein expression of c-Myc and β-catenin in the LS174T and SW480 cell lines. The inhibition rates were 45.14 and 62.06%, respectively, in the LS174T cells (P=0.029 and P=0.003), whereas in the SW480 cells, the rates were 42.32 and 51.48%, accordingly (both P<0.001). Additionally, the protein expression of PP2A was upregulated in the LS174T and SW480 cell lines following *SET* knockdown. The increase rate was 37.55% in the LS174T cells (P=0.012), whereas in theSW480 cells, the rate was 28.39% (P=0.025) (Fig. 3).

## Discussion

SET is a multifunctional protein that is overexpressed in human neoplasms (9,13). The SET protein is a potent PP2A inhibitor that is overexpressed in various human malignancies. Previously, it has been demonstrated that SET upregulation, which leads to PP2A inhibition, is critical for BCR/ABL-positive cells to fulfill their tumorigenic potential (14). Recently, Jiang *et al* (6) reported that *SET* was overexpressed at the mRNA level in 21 tumor samples (70.0%) compared with the corresponding normal tissues. The results of the present study also identified that *SET* expression (at the mRNA level) in 31 patients was markedly increased in 70.9% of tumor specimens compared with adjacent normal tissues. Therefore, these results indicated that *SET* overexpression correlates with colorectal carcinoma progression and that it may play a vital role in the pathogenesis of colorectal cancer. In addition, PP2A was upregulated in 25.8% of samples. PP2A is a widely conserved protein serine/threonine phosphatase that functions as a trimeric protein complex consisting of a catalytic subunit (C or PP2Ac), a scaffold subunit (A or PR65) and one of the alternative regulatory B subunits (15). PP2A plays a crucial role in regulating the cell cycle, signal transduction, cell differentiation, DNA replication and malignant transformation. Previous studies have shown that in target molecules, for which dephosphorylation is critical for the tumor suppressor (16,17), dephosphorylation of the oncogenic transcription factor, c-Myc, is critical for PP2A tumor suppressor activity. Inhibition of PP2A activity induces c-Myc serine 62 (S62) phosphorylation and c-Myc protein stabilization. In addition, it has been reported that c-Myc S62 dephosphorylation inhibits cellular transformation and PP2A-mediated c-Myc dephosphorylation. The c-Myc dephosphorylation is suffice for SV40 small t antigen in human transformation assay activity of PP2A (18). The Wnt/β-catenin signaling pathway often correlates with the overexpression or amplification of the c-Myc oncogene. Paradoxical to the cellular transformation potential of c-Myc is its ability to also induce apoptosis. Notably, c-Myc has been identified as a transcriptional target of the adenomatous polyposis coli (APC)/β-catenin/T-cell factor pathway in colorectal cancer cells (19), suggesting that a method of Wnt signaling function in oncogenesis is through the growth promoting activity of c-Myc (20–22). Our previous study showed that *PP2A* gene expression was increased following *SET* knockdown. Although depletion of *SET* effectively reduced c-Myc S62 protein steady-state levels, *c-Myc* mRNA expression was not significantly decreased by *SET* depletion, implying that *SET* regulates c-Myc S62 protein levels post-transcriptionally through inhibition of PP2A-mediated c-Myc dephosphorylation.

A criticial function of the Wnt pathway is to activate β-catenin-dependent transcription via phosphorylation-regulation. Wnt signaling is transduced through β-catenin, which is regulated by the APC/Axin/glycogen synthase kinase (GSK) 3β complex (23–25). In the absence of Wnt stimulation, GSK-3β constitutively phosphorylates β-catenin at the serine and threonine residues of the NH_2_-terminal region (known as the GSK-3β consensus site), which is well conserved within the catenin family of proteins (26). Phosphorylated β-catenin is subsequently ubiquitinated and degraded through the proteasome pathway. The results of the current study showed that *β-catenin* expression (at the mRNA level) was upregulated in 70.9% of the tumor samples. In addition, the expression (at mRNA and protein level) was significantly decreased following *SET* knockdown. Overall, these results suggested that β-catenin is degraded through phosphorylation via the inhibition of the SET. Previously, Tian *et al* (27) reported that the aberrant expression of the E-cadherin/β-catenin complex is associated with a wide variety of human malignancies and fibrotic disorders. In the present study, E-cadherin expression was significantly downregulated in the LS174T^RNAi^ and SW480^RNAi^ cell lines, suggesting that the suppression of the Wnt signaling pathway in these cells may have resulted from E-cadherin downregulation, which then led to the downregulation of c-Myc expression through the inhibition of the nuclear translocation of β-catenin.

SET is critical for colorectal adenocarcinoma cell growth, since *SET* knockdown by specific siRNA results in significantly promoting apoptosis *in vivo* (6). Previously, Koldobskiy *et al*(28) showed that p53 is a tumor suppressor of which numerous mutations have been found in >50% of malignancies. It has also been found that p53 not only inhibits cell growth, but induces apoptosis. However, in the present study, p53 expression levels were found to significantly decrease in the LS174T^RNAi^ and SW480^RNAi^ cell lines, indicating that this increased cell apoptosis was not directly induced by p53.

In the current study, SET expression at the mRNA and protein level was found to be markedly reduced in LS174T and SW480 *SET*-knockdown cell lines. Furthermore, the expression of PP2A increased and c-Myc levels decreased following *SET* knockdown. In conclusion, these results clearly suggested that *SET* silencing decreases Wnt signaling, indicating that SET plays a crucial role in the Wnt signaling pathway. We hypothesize that SET is a diagnostic marker for prognosis, particularly, neoplasm invasiveness in colorectal cancer. However, future studies are required to determine in detail this correlation and to elucidate the underlying mechanism.

## Figures and Tables

**Table I:** Quantitative real-time polymerase chain reaction primers.

Expression	Primer sequence	Product length, bp
SET	Forward: 5′-GCTCAACTCCAACCACGAC-3′ Reverse: 5′-TCCTCACTGGCTTGTTCATTA-3′	120
PP2A	Forward: 5′-AGTTGGCCAAATGTGTCTCC-3′ Reverse: 5′-GAGTTGCGGTACAAGGAAGG-3′	145
β-catenin	Forward: 5′-GCTGGTGACAGGGAAGACATC-3′ Reverse: 5′-GGTAGTCCATAGTGAAGGCGAAC-3′	116
E-cadherin	Forward: 5′-TTGCTACTGGAACAGGGACACT-3′ Reverse: 5′-GGAGATGTATTGGGAGGAAGGTC-3′	154
c-Myc	Forward: 5′-TCAAGAGGCGAACACACAAC-3′ Reverse: 5′-GGCCTTTTCATTGTTTTCCA-3′	110
p53	Forward: 5′-TAGTGTGGTGGTGCCCTATGAG-3′ Reverse: 5′-AGTGTGATGATGGTGAGGATGG-3′	129
GAPDH	Forward: 5′-GGAAGGTGAAGGTCGGAGT-3′ Reverse: 5′-TGAGGTCAATGAAAGGGGTC-3′	117

PP2A, protein phosphatase 2.

**Table II:** SET, PP2A and β-catenin expression and patient characteristics.

	Gender		Age, years		Dukes’ stage				Differentiation degree		
				
Expression	Male, n	Female, n	≥50	<50	A	B	C	D	Well	Moderate	Poor
Upregulation											
SET	13	9	15	7	1	8	10	3	2	19	1
PP2A	5	3	4	4	0	6	1	1	1	6	1
β-catenin	14	8	15	7	2	9	9	2	2	19	1
Downrugulation											
SET	5	4	6	3	1	6	2	0	2	6	1
PP2A	13	10	16	7	2	8	11	2	3	19	1
β-catenin	13	10	16	7	2	8	11	2	3	19	1

PPA2, protein phosphatase 2.

## References

[1] Bosetti C, Levi F, Rosato V, Bertuccio P, Lucchini F, Negri E, La Vecchia C (2011). Recent trends in colorectal cancer mortality in Europe. Int J Cancer.

[2] Shu Z, Shanrong C (2003). Colorectal cancer epidemiology and prevention study in China. Chin Ger J Clin Oncol.

[3] Chen Y, Zhang YZ, Zhou ZG (2006). Identification of differentially expressed genes in human colorectal adenocarcinoma. World J Gastroenterol.

[4] von Lindern M, van Baal S, Wiegant J, Raap A, Hagemeijer A, Grosveld G (1992). Can, a putative oncogene associated with myeloid leukemogenesis, may be activated by fusion of its 3′ half to different genes: characterization of the set gene. Mol Cell Biol.

[5] Li M, Makkinje A, Damuni Z (1996). The myeloid leukemia-associated protein SET is a potent inhibitor of protein phosphatase 2A. J Biol Chem.

[6] Jiang Q, Zhang C, Zhu J, Chen Q, Chen Y (2011). The SET gene is a potential oncogene in human colorectal adenocarcinoma and oral squamous cell carcinoma. Mol Med Rep.

[7] Van Vlierberghe P, van Grotel M, Tchinda J (2008). The recurrent SET-NUP214 fusion as a new HOXA activation mechanism in pediatric T-cell acute lymphoblastic leukemia. Blood.

[8] Quentmeier H, Schneider B, Röhrs S, Romani J, Zaborski M, Macleod RA, Drexler HG (2009). SET-NUP214 fusion in acute myeloid leukemia and T-cell acute lymphoblastic leukemia-derived cell lines. J Hematol Oncol.

[9] Ouellet V, Le Page C, Guyot MC, LuSSier C, Tonin PN, Proveneher DM, Mes-Masson AM (2006). SET complex in serous epithelial ovarian cancer. Int J Cancer.

[10] Bustin SA, Benes V, Garson JA (2009). The MIQE guidelines: minimum information for publication of quantitative real-time PCR experiments. Clin Chem.

[11] Nolan T, Hands RE, Bustin SA (2006). Quantification of mRNA using real-time RT-PCR. Nat Protoc.

[12] Kim DW, Kim KB, Kim JY, Lee KS, Seo SB (2010). Negative regulation of neuronal cell differentiation by INHAT subunit SET/TAF-Iβ. Biochem Biophys Res Commun.

[13] Cristóbal I, Garcia-Orti L, Cirauqui C, Cortes-Lavaud X, García-Sánchez MA, Calasanz MJ, Odero MD (2012). Overexpression of SET is a recurrent event associated with poor outcome and contributes to protein phosphatase 2A inhibition in acute myeloid leukemia. Haematologica.

[14] Neviani P, Santhanam R, Trotta R (2005). The tumor suppressor PP2A is functionally inactivated in blast crisis CML through the inhibitory activity of the BCR/ABL-regulated SET protein. Cancer Cell.

[15] Janssens V, Goris J (2001). Protein phosphatase 2A: a highly regulated family of serine/threonine phosphatases implicated in cell growth and signalling. Biochem J.

[16] Arroyo JD, Hahn WC (2005). Involvement of PP2A in viral and cellular transformation. Oncogene.

[17] Janssens V, Goris J, Van Hoof C (2005). PP2A: the expected tumor suppressor. Curr Opin Genet.

[18] Arnold HK, Sears RC (2006). Protein phosphatase 2A regulatory subunit B56alpha associates with c-myc and negatively regulates c-myc accumulation. Mol Cell Biol.

[19] Polakis P (2000). Wnt signaling and cancer. Genes.

[20] He TC, Sparks AB, Rago C (1998). Identification of c-myc as a target of the APC pathway. Science.

[21] de La Coste A, Romagnolo B, Billuart P (1998). Somatic mutations of the beta-catenin gene are frequent in mouse and human hepatocellular carcinomas. Proc Natl Acad Sci USA.

[22] Yeh E, Cunningham M, Arnold H (2004). A signalling pathway controlling c-Myc degradation that impacts oncogenic transformation of human cells. Nat Cell Biol.

[23] Willert K, Shibamoto S, Nusse R (1999). Wnt-induced dephosphorylation of axin releases beta-catenin from the axin complex. Genes.

[24] Behrens J, Jerchow BA, Würtele M (1998). Functional interaction of an axin homologconductin, with β-catenin, APC and GSK3β. Science.

[25] Huang H, He X (2008). Wnt/β-catenin signaling: new (and old) players and new insights. Curr Opin Cell Biol.

[26] Bienz M, Clevers H (2000). Linking colorectal cancer to Wnt signaling. Cell.

[27] Tian X, Liu Z, Niu B (2011). E-cadherin/β-catenin complex and the epithelial barrier. J Biomed Biotechnol.

[28] Koldobskiy MA, Chakraborty A, Werner JK (2010). p53-mediated apoptosis requires inositol hexakisphosphate kinase-2. Proc Natl Acad Sci USA.

